# Establishment of an Alternate Care Site (ACS) in Imperial County During COVID-19

**DOI:** 10.5811/westjem.2020.12.49237

**Published:** 2021-03-25

**Authors:** Amelia M. Breyre, Bryan Sloane, Christopher Herring, Howard Backer, Thomas McGinnis, Katherine Staats

**Affiliations:** * University of California San Francisco, Department of Emergency Medicine, San Francisco, California; † Stanford University, Department of Emergency Medicine, Stanford, California;; ‡ Emergency Medical Services/Bioterrorism Preparedness Manager, Imperial County Public Health Department, Imperial, California; § California Emergency Medical Services Authority, Rancho Cordova, California

## Abstract

Imperial County is in southern California, one of the state’s two counties at the international United States-Mexico border. The county is one of the most resource-limited in the state, with only two hospitals serving its 180,000 citizens, and no tertiary care centers. A significant portion of the population cared for at the local hospitals commutes from Mexicali, a large city of 1.2 million persons, just south of Imperial County’s ports of entry. Since May 2020, following an outbreak in Mexicali, Imperial County has seen a significant increase in the number of COVID-19 patients, quickly outpacing its local resources. In response to this surge an alternate care site (ACS) was created as part of a collaboration between the California State Emergency Medical Service Authority (EMSA) and the county. In the first month of operations (May 26–June 26, 2020) the ACS received 106 patients with an average length of stay of 3.6 days. The average patient age was 55.5 years old with a range of 19–95 years. Disposition of patients included 25.5% sent to the emergency department for acute care needs, 1.8% who left against medical advice, and 72.7% who were discharged home or to a skilled nursing facility. There were no deaths on site. This study shares early experiences, challenges, and innovations created with the implementation of this ACS. Improving communication with local partners was the single most significant step in overcoming initial barriers.

## INTRODUCTION

Coronavirus disease 2019 (COVID-19) has created an unprecedented set of medical challenges across the United States that has strained the resources of communities differently. Imperial County is a county in southern California, bordering Mexico. As of early May 2020, there was a significant increase in COVID-19 cases in Mexico and Imperial County. At the time, 25% of COVID-19 tests were positive, representing 834 per 100,000 residents. This was the largest population-adjusted surge in the state of California.^1^ In response to this overwhelming surge in the healthcare system a request from the county was sent to the State of California for additional resources. An Alternate Care Site (ACS) was created as part of a collaboration between the California state Emergency Medical Service Authority (EMSA) and Imperial County. The Imperial County ACS opened on May 25, 2020. This article shares the early experiences with the development of this ACS.

### About Imperial County

Imperial County is bordered in the US by San Diego, Riverside, and Yuma Counties. It is the poorest county in California, and has approximately 180,000 citizens per the 2010 US census. In 2018 the median household income in Imperial County was $45,834, significantly lower than California’s median income ($71,228).^2^ Mexicali is the capital of the state of Baja California in Mexico; it is a city of approximately 1.2 million people along the southern border of Imperial County (Figure).

This border with Mexico represents the fourth busiest pedestrian crossing in the US, with approximately 50,000 individuals passing back and forth daily through three land ports of entry, outside of the COVID-19 pandemic limitations.^3^ A significant number of workers live in Mexico and commute to the US regularly. It is estimated that 10–20% of the population of Mexicali can legally cross the border under the essential worker limitation, approximately 120,000–360,000 persons. Those crossing under these circumstances must be US citizens or lawful permanent residents. At the time of publication, there was no screening or evaluation of COVID-19 at the port of entry. At the time this article was written, Imperial County was a priority in California regarding COVID-19 due to the rapidly increasing numbers of cases, proximity to the outbreak in Baja California, and its resource limitations.

There are two hospitals in Imperial County: El Centro Regional Medical Center (licensed for 161 beds), and Pioneers Memorial Healthcare District (licensed for 107 beds). There are no tertiary care centers within the county – no trauma center, no cardiac catheterization lab, and no stroke center. There is one private EMS 911 transporting agency, and several first-responder public fire departments, most do not transport 911 call patients. There are two air ambulance providers, which are heavily used due to the resource limitations of the county and the number of high-acuity patients. The county routinely collaborates with many state and federal partners including US Customs and Border Protection, US Border Patrol, and land management agencies.

### Alternate Care Sites (ACS)

Alternate care sites is a broad term for temporary sites that are intended to decompress existing healthcare infrastructure by caring for low-acuity patients. These sites are important options to manage medical surge after acute care hospitals have maximized their capacity and capabilities. There are both federal and state guidelines for ACS creation, but the important underlying theme is that an ACS is adaptive to the disaster scenario and the available resources.^4^,^5^ Many different models of ACS have been described.^4^–^8^ In the case of influenza pandemics, ACS can be useful in providing hospital overflow, patient isolation, expanded ambulatory care, care for recovering non-infectious patients, limited supportive care for non-critical patients, primary triage, and rapid patient screening or quarantine.^6^,^7^ Several paradigms advocate for ACS to function as an extension of a supporting hospital, once the hospital exceeds its capacity. In this situation, facilities should not serve as the initial destination for disaster patients as these facilities may lack the appropriate emergency resources. The components of ACS include structure (facilities), stuff (supplies and equipment), staff (personnel), and systems (integrated management policies and processes).^8^

### Objective

The authors of this workgroup represent a variety of stakeholders who were involved in the operations, logistics, planning, and clinical care of patients at the Imperial County ACS. The objective of this study was to shares early experiences, challenges, and innovations created with the implementation of this ACS.

## METHODS

### Site Selection

In Imperial County, the site selected for an ACS was a gymnasium at a local community college, the Imperial Valley College. The site was selected after an assessment conducted by the US Army Corps of Engineers including medical planners, structural, electrical, and civil engineers. The ACS was initially planned as a subacute facility for hemodynamically stable patients with no needs for continuous monitoring, or potential acute decompensation. The facility was set up with supplies from a federal medical cache intended for a skilled nursing facility (SNF) and long-term care facility patients. Patient cots were set up in a large, single room. The overall strategy was to create a facility to offload stable COVID-19 patients and allow hospitals to focus their resources on those with higher acuity medical needs. Given the recent spread of COVID-19 among congregate living sites, such as assisted living facilities, SNFs and multigenerational households, another potential use for the ACS was to quarantine COVID-19 patients to minimize community spread.

### Supplies/Equipment

The facility was able to provide intravenous fluids, low-flow oxygen, and a limited pharmacy formulary. The following resources were not available: single room settings; negative pressure airflow treatment areas; continuous monitoring; or discharge planning. Laboratory services were arranged with both local hospitals on an as-needed basis for patients discharged from those respective facilities. Labs were drawn by ACS medical staff and immediately brought to the hospitals for processing. This allowed for the option of more extensive laboratory services than would have been provided by point-of-care lab testing.

The supplies for our ACS were sourced from federal medical stations (FMS) managed by the Division of Strategic National Stockpile (DSNS), which has rapidly deployable caches containing beds, supplies, and basic medical equipment.^9^–^10^ The FMS supplies were augmented by medical and pharmaceutical supplies obtained from the state of California Emergency Medical Services Authority.

Electrical requirements of the ACS were significant to support multiple biomedical devices running 24 hours a day. The ACS’ need exceeded the initial set-up, and an outside power source from diesel generators was added, with supplementary wiring placed into the COVID patient care area.

### Staff (Personnel)

The initial staffing of the ACS included physicians, advanced practice providers, nurses, pharmacists, paramedics, and emergency medical technicians (EMT). Staffing was supplied through the California Medical Assistance Team (CAL-MAT) program; CAL-MAT is modeled after the federal Disaster Medical Assistance Team. It is based on a volunteer system that is developed and managed by EMSA. Incident command structure was used for personnel management and chain of command.

### ACS Admission Process

Given these resource limitations, the patients selected had to be at low risk for decompensation and semi-ambulatory, ie, able to perform the majority of activities of daily living with minimal assistance. The table lists the inclusion and exclusion criteria. The ACS was available to admit individuals from the two local acute care hospitals as well as licensed SNFs and congregate living facilities.

For potential admissions, the hospital inpatient or emergency department (ED) teams identified patients fulfilling the ACS criteria. A hospital case manager/discharge planner then called the ACS charge nurse to discuss the patient, and if the patient was approved, both a physician-to-physician and nurse-to-nurse sign-out was completed prior to transfer. Patient transport was done through the local and regional transport agency transfer center. Patients arrived with filled prescriptions for home medications and durable medical equipment. On arrival to the ACS, patients received an orientation tour and an identification band, and they were required to sign an ACS agreement. Through a state contract, two of the private agency ambulances were assigned to the ACS to facilitate transfers from the hospitals, and to assist in rapid response for potential patient deterioration within the ACS. The table lists the criteria for transfer to the ED.

## RESULTS

In the first month of operations (May 26–June 17, 2020), the ACS received a total of 106 patients. Of those patients, 54 (50.9%) were male and 52 (49.0%) were female. The average patient age was 55.5 years old with a range of 19–95 years. The most common comorbidities were hypertension (35, 33.0%) and diabetes (39, 36.8%). The average length of stay was a mean of 3.47 days and median of 3 days. The longest length of stay was 16 days. Twenty-seven patients were transferred to the ED (25.5%) for evaluation, with chief complaints ranging from hypotension to worsening hypoxia. Disposition of remaining patients included 1.8% of patients who left against medical advice and 72.7% who were discharged home or to SNF. There were no deaths on site.

## DISCUSSION

### Preliminary Challenges and Solutions

#### Patient Admission

A crucial step after the logistics of establishing an ACS is the recruitment of eligible patients to the new resource from the community. The ACS medical site director contacted local hospital ED and inpatient teams to advocate for the use of the facility. There were two main types of patients targeted: 1) the COVID-19 infected patient admitted to the acute care hospital who was improving but had a continuing oxygen requirement; and 2) the patient with a new COVID-19 diagnosis with a stable oxygen requirement. During the first month of operation, the facility reached a maximum occupancy of 20 patients. At the time this article was written, there was a growing influx of patients as the surge in the county continued and local hospitals became increasingly aware of the ACS capabilities.

In the first month of operations, a total of 106 patients were seen at the ACS with an average length of stay of 3.6 days. This resulted in nearly 300 extra hospital-bed-days of availability to the local county. The success of the program overall, as indicated by patient enrollment, was a product of frequent communication with local hospitals and EDs. Leadership at the ACS regularly advertised their services with local hospitals and continually incorporated feedback as to which services and resources would make the ACS useful to the community.

#### Patients Under Investigation (PUI)

Patients under investigation (PUI) are probable cases with absent or inconclusive laboratory results for COVID-19. These patients were required to have a clinical toxidrome consistent with COVID-19 infection, and findings that also suggested infection. Several patients admitted to the ACS were PUIs, which resulted in a potential exposure risk to themselves, if these patients did not have COVID-19. These PUIs were placed in a well demarcated corner of the open facility, near the entrance to the hot zone, and at least 20 feet from any confirmed positive patient. This PUI section had a dedicated nurse or paramedic provider to minimize PUI exposure to confirmed COVID-19 patients. One provider was assigned to this area on a continuous basis to monitor the patients, and was most often an EMT-Basic. If a patient required a higher level of care of assessment from a provider outside of the PUI zone, the required team member would have to exit the patient care area, partially doff and re-don uncontaminated personal protective equipment (gloves, contact gowns, but not face mask or eyewear) prior to providing care to the PUI patient.

#### Oxygen Supply

Supplemental oxygen is a critical aspect of treatment for COVID-19 patients that can be quickly depleted. Due to the logistical challenges in acquiring and providing a continuous oxygen supply, patient admission criteria to the ACS was limited to an oxygen requirement of ≤ 6 liters per minute. Collaborating with local authorities, we were able to establish a local and renewable source for oxygen tank refills. This became a crucial part of facility sustainability that was initially underestimated.

Oxygen delivery modalities remained a challenge for the ACS. Oxygen concentrators remain the most effective means of sustainable oxygen delivery; however, they were in short supply and their use was limited by electrical requirements. The concentrators also placed a significant strain on the building electrical system, which had not been designed for the uninterrupted demand of a biomedical center. Oxygen regulators were also a scarce resource and required a constant turnover of oxygen tanks. Large, H-cylinder oxygen tanks (> 7000-liter capacity) were also considered. However, due to delivery requiring a hose-based splitting system and a potential tripping hazard limiting patient mobility, the large-cylinder option was deemed too logistically challenging.

#### Home Medications

Given the limits of the onsite pharmacy, patients transferring to the ACS were expected to have their discharge medications in hand when sent from the hospital. However, this proved challenging for patients who did not have health insurance or had financially burdensome copays. We worked with hospital case managers, patient families, and pharmacists to troubleshoot alternative solutions for specific scenarios. For example, patients who were provided albuterol metered-dose inhalers and spacers at the hospital were encouraged to bring those to the ACS. In hindsight, early discussion about the logistics with the home supply of medication would have facilitated smoother transitions of care.

#### Language Barriers

The vast majority of patients admitted to the ACS has been primarily Spanish speaking, which is representative of Imperial County’s population where 85% of the population self-identifies as Hispanic or Latino.^2^ Translating discharge documents, signage for patients and families, and having sufficient numbers of Spanish-speaking staff were necessary adaptations.

#### Patient Care Resources

The majority of patients were discharged from the ACS after being weaned off oxygen and were stable on room air. One patient had to be discharged with a prescription for home oxygen due to a persistent oxygen requirement with exertion. Approximately 30% of patients required transfer back to the local hospitals for escalation of care. The benefit of stat, urgent, and routine laboratory diagnostics may have been beneficial to minimize the number of transfers back to the ED and was further investigated after this preliminary phase. Additionally, in response to requests from local facilities about how we could improve patient care and expand eligible patients, the ACS contracted a physical therapist. Physical therapy provided daily ambulation and leg exercise, assisted in monitoring fitness levels, and provided a presumed decreased risk of venous thromboembolism.

#### Staffing

CAL-MAT and EMSA collaborated to provide staffing to the ACS. Due to the burden of COVID-19 throughout the state of California, finding available clinical providers with acute care or disaster experience was limited. Many of the roles typically filled by a certified nurse assistant or licensed vocational nurse were performed by EMT-Basics based on their ready availability through the CAL-MAT system. Some commonalities among those recruited for the ACS included retired physicians, nurses who were new graduates, or providers who practiced primarily in outpatient settings. The initial staffing structure used a blend of the nurse, paramedic, and EMT role to care for patients that was adjusted for the austere conditions, and generally a different environment than either primarily inpatient or outpatient providers would typically work in. Creating a successful team with the variety of backgrounds and experiences, in the resource-limited setting, was crucial to the success of the mission. Initially it was difficult to anticipate patient volumes, and in the early stages of development, deliberate attempts were made to overstaff the facility. This approach helped tremendously in trying to improve team dynamics, maximize clinical knowledge, and provide flexibility in scheduling.

## LIMITATIONS

There are two noteworthy limitations of this study. Firstly, there is limited outcome data on those patients transferred back to the ED. Unless the patient was returned to the ACS after ED evaluation, it was unknown whether the patient’s condition worsened or improved or there was any additional diagnosis. Secondly, the challenge faced in many disaster medicine scenarios is the limited ability to provide a cost analysis of such interventions. Particularly in a scenario such as the one we report on where there is a collaboration of federal, state, and local resources it can be difficult to calculate the cost benefit of such interventions in a community. Moreover, this ACS was implemented concomitantly with several other health system interventions such as increasing critical care capacity at local hospitals and the development of a regional patient transfer system.

## CONCLUSION

Alternate care sites, by their very nature, are tremendously variable and can be a valuable disaster resource when implemented well. In our recent experience, listening, communicating, and collaborating to meet local community needs is a crucial step in establishing a successful ACS. If there is a mismatch between the requirements of the community and resources provided, then the effort can be expensive, redundant, and ineffective. Once begun, our priority was to adapt to the evolving COVID-19 pandemic in Imperial County. Through our adaptations to the logistical challenges of oxygen distribution, staffing issues, and patient care, we were able to significantly increase the capacity of our ACS, and in turn, serve its local community. We hope this summary of the early stages of development of one ACS, in an area highly impacted by the COVID-19 pandemic, will benefit others in their preparation and response to similar disasters.

## Figures and Tables

**Figure:**
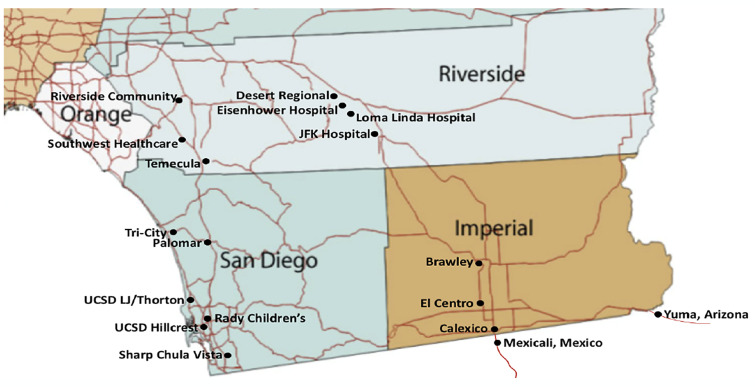
Map of Imperial County in southern California and surrounding geography.

**Table:** Alternate care site (ACS) inclusion, exclusion and immediate transfer to emergency department (ED) criteria.

Inclusion criteria for ACS admission	Exclusion criteria for ACS admission	Criteria for immediate transfer to the ED
Age > 18 years old COVID positive by nasopharyngeal swab within the past month If pregnant, < 20 weeks, and an uncomplicated pregnancy Hemodynamically stable in the last 24 hours, or as approved by an ACS physician. Systolic blood pressure > 90 mm Hg and < 160 mm Hg Diastolic blood pressure > 60 mm Hg and < 110 mm Hg Pulse oximetry > 92% or back to the patient’s prior baseline. Site is capable of 6 liters per minute (LPM) O2 by nasal cannula Heart rate > 60, and < 110 beats per minute Behavioral Cooperative and oriented Able to communicate with medical staff Aware and agrees to ACS conditions Functional Self-feeding Able to get up and ambulate with no more than 1 person assist Low safety risk (falls, wandering, elopement) Able to adhere to rules and be respectful to other patients No significant rehabilitation needs Minor to moderate wound care Heparin lock for IV medications – will leave in for 24 hours	Undifferentiated, potentially life-threatening conditions (eg, chest pain, renal insufficiency) Need for recurrent/frequent lab testing (excluding blood glucose monitoring) Behavioral Severe dementia, delirium or history of sun-downing Acute mental disease Active substance abuse Smoking, vaping (nicotine replacement ok) Hemodialysis unless logistics are established, without increased resource need. Individual isolation (eg, varicella, *C. difficile*, MRSA open wound) Aerosolizing devices such as CPAP/BiPAP, suctioning, oxygen over 15 LPM non-rebreather Need for vital signs more often than every 4 hours for 24 hours Animals	Acute change in oxygen requirement and/or requirement of 6 LPM of oxygen for more than one hour, or any requirement of more than 6 LPM Chest pain or shortness of breath that is new or above the patient’s baseline Increased work of breathing even in the absence of subjective shortness of breath Respiratory rate over 30 breaths per minute Any new neurological symptoms with the exception of generalized weakness, mild dizziness or mild headache Any trauma requiring evaluation Any other expected change or deterioration in condition

*ACS*, alternate care site; *mm Hg*, millimeters mercury; *MRSA*, methicillin-resistant *Staphylococcus aureus*; *CPAP/BIPAP*, continuous airway pressure/bi-level positive airway pressure.
